# Supplementary material for: Elba, a novel developmentally regulated chromatin boundary factor is a hetero-tripartite DNA binding complex
Source: eLife. 2012 Dec 13;1:e00171. doi: 10.7554/eLife.00171 (PMC3510454; doi:10.7554/eLife.00171)
Supplement: Supplementary file 1. — The list of oligonucleotides used in this study. See also ‘Materials and methods’ for the use of each DNA. DOI: http://dx.doi.org/10.7554/eLife.00171.019 [file elife00171s001.doc]

Table Supplement 1: The list of oligonucleotides used in this study.

| Primer | Sequence | Use |
| --- | --- | --- |
| wt-sense3 | 5'-TGC AGC GCC CAA TAA GCA AAT GCA GCG CCC AAT AAG CAA ATG CAG CGC CCA ATA AGC AAA TG-3' | Wildtype Elba binding site x 3 (top) for purification |
| bio-wt-antisense3 | 5'-biotin-AGC ATT TGC TTA TTG GGC GCT GCA TTT GCT TAT TGG GCG CTG CAT TTG CTT ATT GGG CGC TGC A-3' | Wildtype Elba binding site x 3 (bottom) for purification |
| mut3-sense3 | 5'- TGC AGC GCC CCC GAA GCA AAT GCA GCG CCC CCG AAG CAA ATG CAG CGC CCC CGA AGC AAA TG-3' | Mutant Elba binding site x 3 (top) for purification |
| bio-mut3-antisense3 | 5'-biotin-AGC ATT TGC TTC GGG GGC GCT GCA TTT GCT TCG GGG GCG CTG CAT TTG CTT CGG GGG CGC TGC A-3' | Mutant Elba binding site x 3 (bottom) for purification |
| BamHI-CG12205-03 | 5'-ATG GAT CCC AAT GAT TAA CCG CCG TC-3' | PCR of Elba1 protein-coding region |
| SalI-CG12205-04 | 5'-CTG TCG ACT ACT TTG ATT TGC GCT GC-3' | PCR of Elba1 protein-coding region |
| BamHI-CG9883-01 | 5'-ATG GAT CCA AAT GGC CGG CGG ATT TC-3' | PCR of Elba2 protein-coding region |
| SalI-CG9883-02 | 5'-GCG TCG ACT GCT AAT ACT CCT CCT TG-3' | PCR of Elba2 protein-coding region |
| BamHI-CG15634-01 | 5'-CAG GAT CCC CAT CAT GAG CAA CAT CAG C-3' | PCR of Elba3 protein-coding region |
| XhoI-CG15634-02 | 5'-GTC TCG AGG CCA ACT AAT GAA GTT CGT C-3' | PCR of Elba3 protein-coding region |
| BamHI-CG3227-01 | 5'-CAG GAT CCG AGG ATT GAA CAT GGA AAA CC-3' | PCR of Insv protein-coding region |
| XhoI-CG3227-02 | 5'-GTC TCG AGG GAT TGT GAG AGT TTG TGC G-3' | PCR of Insv protein-coding region |
| CG3227-04 | 5'-CTG CTA CAC GTT AGT TCG-3' | Reverse-transcription of *insv* |
| HA-sense | 5'-CTA GAC CAT GGT CTA TCC TTA TGA CGT CCC TGA CTA TGC CAG CCT TGG-3' | HA tag of pKS(+)HA (sense strand) |
| HA-antisense | 5'-GAT CCC AAG GCT GGC ATA GTC AGG GAC GTC ATA AGG ATA GAC CAT GGT-3' | HA tag of pKS(+)HA (antisense strand) |
| XhoI-HA-upstream | 5'-ACC TCG AGT ATC CTT ATG ACG TCC CTG-3' | PCR of HA tag-attached cDNA for pET15b constructs |
| FLAG-sense | 5'-CTA GAC CAT GGA CTA CAA GGA CGA CGA TGA CAA GGG-3' | FLAG tag of pKS(+)FLAG (sense strand) |
| FLAG-antisense | 5'-GAT CCC CTT GTC ATC GTC GTC CTT GTA GTC CAT GGT-3' | FLAG tag of pKS(+)FLAG (antisense strand) |
| GST-met | 5'-CGA AGA TCT CTA TGT CCC CTA TAC TAG-3' | PCR of the GST tag for pKS(+)FLAG-GST |
| pGEX-reverse | 5'-CCG GGA GCT GCA TGT GTC AGA GG-3' | PCR of the GST tag for pKS(+)FLAG-GST |
| SESAqPCR_for03-2 | 5'-AGA GGA AGA GAG CGG AAA GTG-3' | qPCR of *Fab-7* pHS1 locus in ChIP |
| SESAqPCR_rev03 | 5'-CGT TGA TAT GCC CCA ATG TT-3' | qPCR of *Fab-7* pHS1 locus in ChIP |
| twein-real-F2 | 5'-TCG AAT CGA TCC ACC TGC CAT-3' | qPCR of *twe* locus in ChIP |
| twein-real-R2 | 5'-TTG GGC AGC CAT CGT TTC TCA TTG-3' | qPCR of *twe* locus in ChIP |
| Sxl+1171_for | 5'-GTG GTT ATC CCC CAT ATG GC-3' | qPCR of *Sxl* locus in ChIP |
| Sxl+1171_rev | 5'-GCC AAA GAG GTA TGG GTA GC-3' | qPCR of *Sxl* locus in ChIP |
| T7-CG11205-forward | 5'-TAA TAC GAC TCA CTA TAG GGA TTA ACC GCC GTC AGC GTT TG-3' | Elba1 RNAi |
| T7-CG11205-reverse | 5'-TAA TAC GAC TCA CTA TAG GGA TGG TGT TCC TCA TCA GCT GC-3' | Elba1 RNAi |
| T7-CG9883-forward | 5'-TAA TAC GAC TCA CTA TAG GGA TTT CGA CCG TAC GAT CAG GAC-3' | Elba2 RNAi |
| T7-CG9883-reverse | 5'-TAA TAC GAC TCA CTA TAG GGA ACT GGC TTA ACT GGC TGG TTG-3' | Elba2 RNAi |
| T7-CG15634-forward | 5'-TAA TAC GAC TCA CTA TAG GGA GCA ACA TCA GCA ACG ACA GTG-3' | Elba3 RNAi |
| T7-CG15634-reverse | 5'-TAA TAC GAC TCA CTA TAG GGA GGC GCT CGC CCG CCT TTT CAT TC-3' | Elba3 RNAi |
| T7-CG3227-forward | 5'-TAA TAC GAC TCA CTA TAG GGA TTC GCA GGC AAG GAG GAG CAA G-3' | Insv RNAi |
| T7-CG3227-reverse | 5'-TAA TAC GAC TCA CTA TAG GGA GGT GCT CAG GCT GTT CAG ACT C-3' | Insv RNAi |

**Table Supplement 1. The list of oligonucleotides used in this study**. See also 'Materials and Methods' part for the use of each DNA.
